# Assessment of CYP1A2 enzyme activity in relation to type-2 diabetes and habitual caffeine intake

**DOI:** 10.1186/s12986-016-0126-6

**Published:** 2016-10-06

**Authors:** Emily Urry, Alexander Jetter, Hans-Peter Landolt

**Affiliations:** 1 Institute of Pharmacology and Toxicology, University of Zürich, Winterthurerstrasse 190, 8057 Zürich, Switzerland; 2 Zürich Center of interdisciplinary Sleep Research, University of Zürich, Zürich, Switzerland; 3 Department of Health Sciences and Technology, ETH Zürich, Zürich, Switzerland; 4 Department of Clinical Pharmacology and Toxicology, University Hospital Zürich, Zürich, Switzerland

**Keywords:** Caffeine, Paraxanthine, Phenotyping, HPLC

## Abstract

**Background:**

Coffee consumption is a known inducer of cytochrome P450 1A2 (CYP1A2) enzyme activity. We recently observed that a group of type-2 diabetes patients consumed more caffeine (coffee) on a daily basis than non-type-2 diabetes controls. Here, we investigated whether type-2 diabetes cases may metabolize caffeine faster than non-type-2 diabetes controls.

**Methods:**

To estimate CYP1A2 enzyme activity, an established marker of caffeine metabolism, we quantified the paraxanthine/caffeine concentration ratio in saliva in 57 type-2 diabetes and 146 non-type-2 diabetes participants in a case–control field study. All participants completed validated questionnaires regarding demographic status, health and habitual caffeine intake, and were genotyped for the functional -163C > A polymorphism of the *CYP1A2* gene.

**Results:**

In the diabetes group, we found a larger proportion of participants with the highly inducible *CYP1A2* genotype. Furthermore, the paraxanthine/caffeine ratio, time-corrected to mitigate the impact of different saliva sampling times with respect to the last caffeine intake, was higher than in the control group. Participants who reported habitually consuming more caffeine than the population average showed higher CYP1A2 activity than participants with lower than average caffeine consumption. Multiple regression analyses revealed that higher caffeine intake was potentially an important mediator of higher CYP1A2 activity.

**Conclusions:**

Estimated CYP1A2 enzyme activity, and thus speed of caffeine metabolism, was higher in our type-2 diabetes group; this was possibly due to higher intake of caffeine, a known inducer of CYP1A2 enzyme activity. Given the fairly small sample sizes, the results need to be considered as preliminary and require validation in larger populations.

**Electronic supplementary material:**

The online version of this article (doi:10.1186/s12986-016-0126-6) contains supplementary material, which is available to authorized users.

## Background

Caffeine is almost completely metabolized in the body by cytochrome P450 1A2 (CYP1A2). This enzyme accounts for the metabolism of caffeine to its principal metabolite, paraxanthine [1]. In vivo, CYP1A2 activity exhibits a significant degree of inter-individual variation (see [2] for review). Inter-individual variability in CYP1A2 enzyme activity is typically between 5- and 15-fold in healthy humans [3, 4], possibly due to environmental and genetic factors. For example, coffee consumption and cigarette smoking both induce CYP1A2 activity, in a dose-dependent manner [4]. Interestingly, rodent models demonstrate that the blood-glucose-regulatory hormone insulin also acts as an inducer of CYP1A2 activity [5]. While this relationship has not been directly assessed in humans, a correlational study revealed a positive relationship between CYP1A2 activity and endogenous insulin levels in premenopausal women [6]. Also functional variations in the *CYP1A2* gene may contribute to inter-individual differences in enzyme activity [7]. Indeed, a single nucleotide polymorphism (SNP) (−163C > A) of *CYP1A2* has been associated with increased enzymatic activity in smokers [8].

Systemic caffeine clearance is considered the gold-standard approach to estimating CYP1A2 activity [9], which reflects the combined effects of genetic, environmental and endogenous factors [10]. However, this method requires extensive blood sampling, which is expensive, invasive and time consuming [2]. A validated alternative is to determine the concentration ratio of paraxanthine to caffeine in a saliva sample collected 6 h post caffeine dose [9, 11].

In a case–control field study, we recently found that type-2 diabetes patients consumed more caffeine than non-type-2 diabetes controls, possibly to attenuate daytime sleepiness typically associated with the disease [12]. Based on the above presented evidence that caffeine and insulin act as possible inducers of CYP1A2 activity, we hypothesized that the type-2 diabetes patient group would show higher CYP1A2 enzyme activity than the non-type-2 diabetes control group. To our knowledge, only one study has previously assessed CYP1A2 enzyme activity in type-2 diabetes patients, and no difference was found between case and control groups [13]. The findings, however, are limited by the small sample size (*n* = 16 patients and controls), the long time frame between caffeine ingestion and provision of saliva (8 h) [9, 11], and the lack of data regarding habitual caffeine/coffee intake, smoking, and *CYP1A2* genotype. All these factors may impact CYP1A2 activity in patients and controls.

## Methods

### Subjects

A total of 445 study participants were recruited. Two type-1 diabetes participants were excluded due to the different pathophysiology of type-1 and type-2 diabetes; 7 participants of non-European descent were excluded due to genetic variation between populations of different ethnic origin. In addition, 179 participants who did not report a 3–12 h time interval between their final caffeine portion and saliva sampling, were excluded. This is because the paraxanthine/caffeine ratio only reliably measures CYP1A2 enzyme activity when there is a 3–12 h time interval between caffeine intake and provision of the saliva sample, due to the non-linear kinetics of caffeine metabolism [14]. Two participants were excluded due to missing saliva. In 17 participants, the HPLC measurements were below the quantification limit (BQL) of caffeine (BQL = 0.077 μg/ml; *n* = 5), paraxanthine (BQL = 0.024 μg/ml; *n* = 5) or both analytes (*n* = 7); and in 23 participants, the corrected paraxanthine/caffeine ratio was negative (see below). Finally, 14 participants were excluded because of technical difficulties with the HPLC quantification. The final sample comprised 203 participants (57 type-2 diabetes cases and 146 non-type-2 diabetes controls).

The type-2 diabetes status was determined by an affirmative response to the question: “Over the past 12 months, have you suffered from type-2 diabetes?”. As well as, an answer to the question: “If you suffer from type-2 diabetes, when did you receive your diagnosis?”; and a report of a diabetes-appropriate treatment regime of oral medication and/or insulin.

### Questionnaire assessment

Questionnaires gathered information regarding demographic status, health and habitual caffeine intake. The survey was completed either online (2ask^®^ survey software) or in paper form. Habitual caffeine intake was assessed using an extended version of the caffeine intake questionnaire of the sleep laboratory of the University of Zürich [15]. Participants were asked to report how frequently (per day or per week) they usually consumed a given range of caffeine-containing foods, drinks, medications and supplements. Additional file 1: Table S1 displays the estimated caffeine content (mg/serving) of each item in the questionnaire. These data were used to calculate participants’ daily habitual caffeine intake.

### Saliva sampling

Participants gave two samples of saliva at home and then posted them back to the laboratory in a pre-paid envelope (Tyvek^®^ material; DuPont). Beforehand, participants were posted a parcel containing detailed information, a checklist (to record time/date of sampling and caffeinated products consumed that day), and two saliva receptacles [*1)* Salivette^®^ swab (Sarstedt, Nümbrecht, Germany) to determine caffeine and paraxanthine concentrations; *2)* Oragene^®^ DNA kit (DNA Genotek Inc., Ottawa, Canada) for DNA extraction and genotyping]. Participants were instructed to give both saliva samples at bedtime, and without eating, drinking, chewing gum or smoking in the thirty minutes beforehand; and also to complete the checklist. Contact details of the research team were available in case participants needed assistance.

### Genomic assessment with salivary DNA

Oragene receptacles were stored at room temperature until genomic DNA was extracted from saliva according to DNA Genotek Inc.’s instructions. Participants were genotyped for the functional rs762551 polymorphism of the *CYP1A2* gene, a demonstrated determinant of inducible CYP1A2 activity [8, 16], and labelled ‘highly inducible’ or ‘less inducible’ caffeine metabolizers (A/A genotype = ‘highly inducible’; A/C and C/C genotypes = ‘less inducible’). All genetic analyses were replicated at least once for independent confirmation of the results. Experimental protocols are described in Additional file 1.

### HPLC assessment of salivary caffeine and paraxanthine

The saliva samples were delivered to the laboratory at room temperature. Upon receipt, the salivettes were stored immediately at −20 °C. The stability of salivary caffeine and paraxanthine concentrations over 14 days at room temperature has previously been confirmed [17].

After thawing, saliva was extracted from the Salivette^®^ according to the manufacturer’s instructions (centrifugation for 2 min at 1,000 g). Salivary caffeine and paraxanthine concentrations were quantified by HPLC, coupled to a UV detector, essentially as described by Fuhr and Rost [11], but with minor modifications as described in Additional file 1.

The stability of salivary caffeine and paraxanthine concentrations during long-term storage at −20 °C was confirmed in a sub-sample (*n* = 7). Saliva was analyzed at two time points, ten months apart. Statistical comparisons revealed that there was no significant difference between the caffeine concentrations at the two time points: 2.590 ± 1.573 (SD) vs. 2.523 ± 1.351 μg/ml (*p* > 0.8; paired-sample *t*-test). There was also no significant difference between the paraxanthine concentrations at the two time points: 0.920 ± 0.461 (SD) vs. 0.789 ± 0.341 μg/mL (*p* > 0.2).

### Determination of corrected paraxanthine/caffeine ratio

The present field study participants reported varied caffeine consumption on the day of saliva sampling, and varied time intervals between their last caffeine intake and the saliva sampling. While Perera and colleagues [18] demonstrated that CYP1A2 activity can be reliably assessed without a 24-h period of caffeine abstinence, assessment of CYP1A2 phenotypes is very time-dependent [19–22]. Correlation analyses between immunoreactive CYP1A2 in the liver, intrinsic clearance for caffeine-3-demethylation to paraxanthine, and various plasma, saliva, and urine based CYP1A2 metrics showed that the saliva paraxanthine/caffeine ratio 6 h after caffeine intake had the best correlation to intrinsic caffeine-to-paraxanthine clearance, which is the “gold standard” for CYP1A2 activity assessment [9]. That is, six hours post caffeine dose, the molar concentration ratio of salivary paraxanthine to caffeine provides the most valid estimate of CYP1A2 enzyme activity [9, 11]. We therefore developed a method to adjust the CYP1A2 activity ratio values to the optimal 6-h post-dose sampling time point, and to thus allow direct comparison within and between groups.

**Fig. 1:**
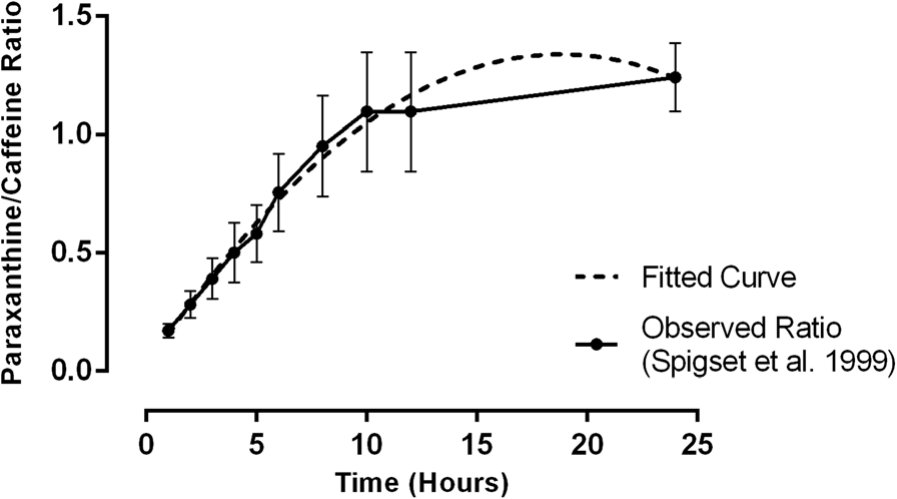
Relationship between sampling time and salivary paraxanthine/caffeine ratio. Solid line: mean observed ratio estimates based on data of Spigset et al. [14]. Error bars show standard deviation across the mean of observed ratio data (*n* = 12). Dotted line: ratio based on fitted curve (dotted line) using a second-order polynomial model: *Y = A + (B x X) + (C x X* ^*2*^ *)*. Best-fit values (95 % confidence intervals): *A = 0.016 (-0.206 - 0.238)*; *B = 0.141 (0.090 - 0.191)*; *C = -0.004 (-0.006 - -0.002)*. Equation: *Y = 0.016 + (0.141 x X) + (-0.004 x X* ^*2*^ *)*; Y = paraxanthine/caffeine ratio; X = time interval between final caffeine intake and saliva sampling

Spigset and colleagues [14] investigated the relationship between sampling time and individual salivary paraxanthine/caffeine ratios, after intake of a single oral dose of 200 mg caffeine, in 12 healthy, young men in a controlled, laboratory setting. Based on inspection of Fig. 1 in their publication, the lowest and highest paraxanthine/caffeine ratio, at each time point, was recorded. The mean of the lowest and highest ratio was then calculated and entered in GraphPad Prism (La Jolla, California, USA). After fitting a curve to the data set, the equation y = 0.016 + (0.141 * x) + (−0.004 * x ^*2*^ *)* was used to estimate the participants’ paraxanthine/caffeine ratio (‘y’), if the time span between last caffeine intake and provision of saliva (‘x’) was known. A time span of 6 h equates to a mean paraxanthine/caffeine ratio of 0.725.

**Fig. 2:**
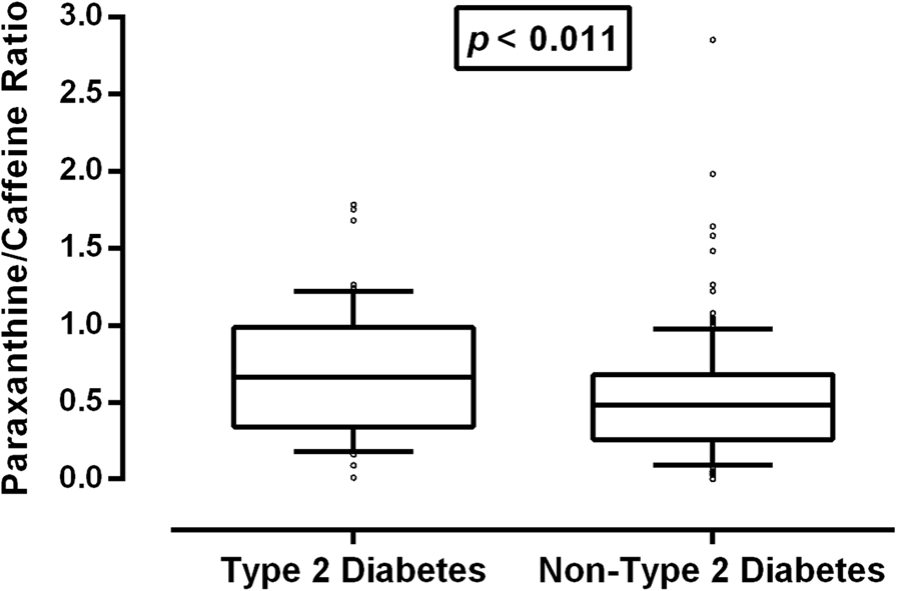
Paraxanthine/caffeine ratios in type-2 diabetes patient and non-type-2 diabetes control groups. Boxplots represent paraxanthine/caffeine ratios corrected to an “ideal” time interval between last caffeine intake and saliva sampling of 6 h (box: 25^th^ percentile, median and 75^th^ percentile; whiskers = 10^th^ to 90^th^ percentiles; dots: individual data points outside of the whisker range). Statistics compared type-2 diabetes patient (*n* = 57) and non-type-2 diabetes control (*n* = 146) groups by independent samples *t*-test on square-rooted data (2-tailed; equal variances assumed). Statistical analysis with the non-parametric Mann-Whitney U-test on non-transformed corrected paraxanthine/caffeine ratios confirmed the robustness of the result: T2D vs. non-T2D: mean rank 120.93 vs. 94.61; exact sig. 2-tailed: *p* = 0.004)

Figure 2 shows the relationship between the time interval between caffeine intake and saliva sampling, and the paraxanthine/caffeine ratio for both the mean observed ratios based on the data of Spigset et al. [14], and the ratio based on the fitted curve using the above-mentioned equation. The fitted curve explained roughly 70 % (R^2^ = 0.702) of the variance in the observed ratio data.

To estimate the participants’ paraxanthine/caffeine ratio adjusted to the ‘ideal’ time interval of 6 h, several steps were taken. First, based on the molar concentrations of paraxanthine and caffeine, the ‘actual’ paraxanthine/caffeine ratio was calculated for each participant. Next, the Spigset-derived equation was used to estimate the ‘correct’ mean ratio, based on participants’ reported time interval between caffeine intake and saliva sampling. The difference between the ‘actual’ ratio and the ‘correct’ mean ratio was then calculated. Finally, participants’ time-corrected paraxanthine/caffeine ratio, to assume a 6 h ‘ideal’ time span, was determined by adding this difference to the equation’s estimate of the paraxanthine/ caffeine ratio at a 6-h time interval (0.725). Graphically spoken, the individual ratio value was shifted on a curve with the slope of the Spigset data-derived mean curve to the ideal time of 6 h. The paraxanthine/caffeine ratio data, derived from our time-correction technique, ranged from 0.00 to 2.85 (mean: 0.577 ± 0.411 [SD]; *n* = 203).

### Statistical analyses

Analyses were performed with Microsoft Excel 2010 (Microsoft Corp., Seattle, USA) and IBM SPSS Statistics 22 (IBM Corp., Armonk, USA), and adhered to documented statistical principles [23]. Mean values (± standard deviations) of raw data are reported and significance was set at α < 0.05. Continuous variables that were not normally distributed (based on visual inspection of histogram and SPSS-derived skewness score −1 > *x* <1) were transformed to approximate a normal distribution. Table legends indicate the successful transformation method. If data were missing for a variable, the smaller sample size for that variable was reported with the results. Data from type-2 diabetes and non-type-2 diabetes groups were compared by Fisher’s Exact Test (nominal data) and independent samples *t*-test (normally distributed continuous data). Independent samples *t*-tests were used to compare the corrected paraxanthine/caffeine ratio data grouped by variables that were previously reported to influence CYP1A2 enzyme activity, including age (≤ mean age of 59.3 years vs. > mean age), body mass index (underweight/healthy ≤ 24.9 vs. overweight/obese > 24.9), habitual caffeine intake [lower/normal habitual caffeine intake (≤ Swiss average of 288 mg/day) vs. higher habitual caffeine intake (> Swiss average)], contraceptive pill (no vs. yes), *CYP1A2* -163C > A genotype (A/C and C/C allele carriers vs. A/A allele carriers), gender (male vs. female), insulin administration (no vs. yes), long-term medication (no vs. yes) and smoking (non smoking vs. smoking). Within the type-2 diabetes group, the corrected paraxanthine/caffeine ratio data was compared between selected binary covariates (habitual caffeine intake, CYP1A2 inducibility, gender, insulin administration, smoking) by independent samples *t*-test on raw data that approximated a normal distribution.

If there was a statistical difference in the results of the independent samples t-tests across the whole sample, which compared the corrected ratio data grouped by variables previously reported to influence CYP1A2 activity, then that covariate was included in a multiple regression analysis (simultaneous entry). The regression model tested the association between the selected variables and the paraxanthine/caffeine ratio across the whole group. The outcome variable was the corrected paraxanthine/caffeine ratio, transformed by square root to approximate a normal distribution. The two predictors were type-2 diabetes status (nominal; binary) and high (> Swiss average of 288 mg/day) caffeine intake (nominal; binary). ‘Insulin’ was not included as a predictor variable since it was only administered by type-2 diabetes patients, and thus its inclusion would confound the results. The model was tested to ascertain that it met the statistical assumptions of multiple regression [23, 24].

## Results

Table 1 reports the characteristics of the type-2 diabetes cases and the non-type-2 diabetes controls. The groups differed in age, gender, body mass index, long-term medication intake and oral contraceptive use.

**Table 1:** Characteristics of whole sample, and split by type-2 diabetes and non-type-2 diabetes group [continuous variables: mean (± standard deviation); categorical variables: frequency (% of total)]

Variable		Whole sample (*n* = 203)	Type-2 diabetes cases (*n* = 57)	Non-type-2 diabetes controls (*n* = 146)	*p*-value between groups
Age (years)		59.3 (±15.9)	63.9 (±9.9)	57.4 (±17.4)	0.008
Male Gender (%)		87 (42.9 %)	38 (66.7 %)	49 (33.6 %)	<0.001
Body Mass Index (BMI; kg/m^2^)^a^		25.1 (±4.5)	28.6 (±5.3)	23.7 (±3.3)	<0.001
Overweight/Obese BMI (%)^b^		84 (41.8 %)	42 (73.7 %)	42 (29.2 %)	<0.001
Smoking (% yes)		21 (10.3 %)	9 (15.8 %)	12 (8.2 %)	0.127
Alcohol Intake (% yes)^c^		78 (38.4 %)	16 (28.1 %)	62 (42.5 %)	0.077
Long-Term Medication (% yes)		129 (63.5 %)	55 (96.5 %)	74 (50.7 %)	<0.001
Oral Medication for Diabetes (% yes)		42 (20.7 %)	42 (73.7 %)	0 (0 %)	<0.001
Insulin Injections for Diabetes (% yes)		19 (9.4 %)	19 (33.3 %)	0 (0 %)	<0.001
Contraceptive Pill (% yes)		9 (4.4 %)	0 (0 %)	9 (6.2 %)	0.064
Total Habitual Caffeine Intake (mg/day)^d^		295.8 (±158.1)	365.2 (±191.3)	268.7 (±134.4)	<0.001
Caffeine from Coffee (mg/day)^d, e^		240.3 (±162.2)	306.9 (±195.7)	214.3 (±139.4)	0.001
Higher Habitual Caffeine Intake (% yes)^f^		64 (31.5 %)	28 (49.1 %)	36 (24.7 %)	0.001
Salivary Caffeine Concentration (μmol/l)^d^		11.0 (±7.7)	11.9 (±8.2)	10.6 (±7.6)	0.259
Salivary Paraxanthine Concentration (μmol/l)^d^		5.2 (±3.4)	6.0 (±3.4)	4.9 (±3.4)	0.024
Time between saliva sample and final caffeine portion (h)		6.7 (±2.6)	5.8 (±2.6)	7.1 (±2.5)	0.001
Gene Cytochrome P450-1A2 (*CYP1A2*)^g^					
Allele Frequency (%)	A	276 (69.0 %)	83 (74.1 %)	193 (67.0 %)	0.186
	C	124 (31.0 %)	29 (25.9 %)	95 (33.0 %)	
Genotype Frequency (%)					0.036
	A/A	97 (48.5 %)	34 (60.7 %)	63 (43.8 %)	0.040
	C/A	82 (41.0 %)	15 (26.8 %)	67 (46.5 %)	0.011
	C/C	21 (10.5 %)	7 (12.5 %)	14 (9.7 %)	0.610
Enzyme Inducibility (%)	High	97 (48.5 %)	34 (60.7 %)	63 (43.8 %)	0.040
	Less	103 (51.5 %)	22 (39.3 %)	81 (56.3 %)	

*Abbreviations T2D* type-2 diabetes, *Non-T2D* non-type-2 diabetes, Data for continuous variables are means (± standard deviation) of raw data. *P*-values (2-tailed) were calculated using independent samples t-tests, comparing T2D and Non-T2D groups, on raw data. If raw data was abnormally distributed, the data was transformed to achieve a normal distribution before the *t*-test was applied (method of transformation noted in legend). Data for categorical variables are frequencies (%). *P*-values (exact; 2-tailed) were calculated using Fisher’s exact test

^a^Raw data transformation: Log10; T2D (*n* = 57); Non-T2D (*n* = 144). ^b^Overweight/Obese BMI >24.9 vs. Underweight/Healthy BMI ≤24.9; T2D (*n* = 57); Non-T2D (*n* = 144). ^c^Consume 3 or more alcoholic drinks per week. ^d^Raw data transformation: Square root. ^e^Includes caffeine from decaffeinated coffee (4.5 mg/cup). ^f^Higher habitual caffeine intake (> Swiss average of 288 mg/day) vs. Lower/Normal habitual caffeine intake (≤ Swiss average). ^g^SNP ID: rs762551. T2D (*n* = 56); Non-T2D (*n* = 144). Highly inducible = genotype A/A. Less inducible = genotypes A/C and C/C

The total self-reported habitual caffeine intake was 96.5 mg higher per day in the type-2 diabetes group (Table 1). Coffee was the major source of caffeine for both groups. Despite the shorter time interval between saliva sampling and the final portion of caffeine intake in the patients, the mean salivary concentration of paraxanthine was significantly higher in the type-2 diabetes patient group than in the control group (Table 1).

The -163C > A allele frequencies of the *CYP1A2* gene were similar in type-2 diabetes and non-type-2 diabetes groups (Table 1). However, compared to the control group, there was a higher proportion of diabetes participants with the highly-inducible A/A genotype and a lower proportion of diabetes participants with the less-inducible A/C and C/C genotypes (Table 1).

As illustrated in Fig. 1, the mean time-corrected paraxanthine/caffeine ratio was significantly higher in type-2 diabetes cases than in non-type-2 diabetes controls (type-2 diabetes patients: 0.700 ± 0.426; non-type-2 diabetes controls: 0.529 ± 0.396; *p* = 0.010, two-tailed *t*-test). This finding indicates a higher mean CYP1A2 enzyme activity in the group of type-2 diabetes patients. When those patients who reported to take insulin were excluded, the difference was no longer significant (0.664 vs. 0.529; *p* = 0.121). Indeed, CYP1A2 enzyme activity was significantly faster in participants who administered insulin (Table 2). When only type-2 diabetes patients were assessed, however, the difference was not significant (0.664 vs. 0.770; *p* = 0.382).

**Table 2:** Independent samples t-tests comparing time-corrected paraxanthine/caffeine ratios by age, body mass index, caffeine intake, contraceptive pill, *CYP1A2* inducibility, gender, insulin administration, long-term medication, smoking status

	Group	N	Time-corrected paraxanthine/caffeine ratio	*p* value
Age	≤59.3 years	77	0.604 (±0.364)	0.290
	>59.3 years	126	0.561 (±0.438)	
BMI	≤24.9 kg/m^2^	117	0.560 (±0.415)	0.580
	>24.9 kg/m^2^	84	0.603 (±0.408)	
Caffeine Intake	≤288 mg/day	139	0.535 (±0.431)	0.010
	>288 mg/day	64	0.669 (±0.350)	
Contraceptive Pill	No	194	0.579 (±0.417)	0.979
	Yes	9	0.545 (±0.282)	
*CYP1A2* Inducibility	Less (C/A and C/C genotypes)	103	0.552 (±0.430)	0.284
	High (A/A genotype)	97	0.609 (±0.394)	
Gender	Male	87	0.631 (±0.467)	0.237
	Female	116	0.537 (±0.360)	
Insulin Administration	No	184	0.557 (±0.414)	0.016
	Yes	19	0.770 (±0.336)	
Medication	No	74	0.510 (±0.311)	0.187
	Yes	129	0.616 (±0.456)	
Smoking Status	Non Smoking	182	0.573 (±0.410)	0.896
	Smoking	21	0.617 (±0.430)	

Values are given as mean (±SD). Independent samples t-tests were applied to time-corrected paraxanthine/caffeine ratios transformed by square root to approximate a normal distribution. Statistical data reported assumed equal variances. *P*-values reflected a 2-tailed test. Results are reported to 3 decimal places

It is estimated that the Swiss population consumes roughly 288 mg caffeine per capita and day [12, 25]. Participants who reported habitually consuming higher amounts of caffeine (>288 mg/day), showed significantly faster CYP1A2 enzyme activity, compared to participants consuming less caffeine (≤288 mg/day) (Table 2). Within the type-2 diabetes sample, patients who reported habitually consuming more caffeine than the population average showed a numerically faster CYP1A2 activity (0.763 vs. 0.638), but not to a significant degree (*p* = 0.276).

The CYP1A2 genotype, gender and smoking status had no significant effect on the mean time-corrected paraxanthine/caffeine ratio (Table 2).

Multiple regression analysis was used to predict the paraxanthine/caffeine ratio across the whole sample (Table 3). The two selected predictors of CYP1A2 enzyme activity, i.e., type-2 diabetes status and higher caffeine intake (see Methods), significantly predicted the paraxanthine/caffeine ratio (F_2,200_ = 5.580, *p* = 0.004). While they accounted for only 5.3 % of the variation in the data, both made statistically significant contributions to the prediction (T2D status: Beta = 0.146; *p* = 0.040; higher caffeine intake: Beta = 0.146; *p* = 0.041). When the regression model was run with caffeine intake as a continuous variable, the model also predicted the paraxanthine/caffeine ratio (ANOVA: F_2,200_ = 5.298, *p* = 0.006), with type-2 diabetes being a significant predictor (Beta = 0.145; *p* = 0.044) and caffeine intake exhibiting a strong trend to predict the ratio (Beta = 0.137; *p* = 0.056).

**Table 3:** Multiple regression analysis to predict the paraxanthine/caffeine ratio (*N* = 203)

Model summary	Covariates	Unstandardized coefficients		Standardized coefficients	*p* value
		B	Std. error	Beta	
	T2D status (Ref: non-T2D)	0.088	0.043	0.146	0.040
	Higher caffeine intake *(Ref: no)*	0.085	0.041	0.146	0.041
R^2^	0.053				
Adjusted R^2^	0.043				
Model ANOVA	*p* = 0.004				

*Abbreviations*: *Ref* reference, *T2D* type-2 diabetes, *Non-T2D* non-type-2 diabetes

Table represents multiple regression analysis to predict the corrected paraxanthine/caffeine ratio. The 2 predictor variables were entered simultaneously. Continuous variables were raw data transformed by square root to achieve a normal distribution (corrected paraxanthine/caffeine ratio). Categorical variables were binary (T2D status, higher caffeine intake). [‘Higher’ caffeine intake > 288 mg/day (Swiss daily average caffeine intake)]

## Discussion

In support of our hypothesis, the main finding of this field study was that CYP1A2 enzyme activity was significantly higher in a type-2 diabetes group compared to a control group. Since caffeine is almost completely metabolized by CYP1A2 [1], this faster enzyme activity indicates a faster metabolism of caffeine in the type-2 diabetes participants. Indeed, patients’ salivary concentrations of paraxanthine, caffeine’s major metabolite [1], were significantly higher at bedtime. The results indicate that the previously described inducing effect of caffeine on its own CYP1A2-mediated metabolism may also be present in type-2 diabetes patients.

We used a novel correction technique to adjust participants’ paraxanthine/caffeine ratios, an established marker of CYP1A2 enzyme activity, to account for the varied reported time intervals between last caffeine intake and saliva sampling. Based on the equation we derived from published data [14], the paraxanthine/caffeine ratio of the present participants was adjusted to reflect a ratio that would stem from the ‘ideal’ time interval of 6 h [9, 11]. The time-corrected paraxanthine/caffeine ratios obtained with this method were comparable to published reports [11], which supports the validity of our approach.

In accordance with previous research [4], participants who habitually consumed higher amounts of caffeine showed higher paraxanthine/caffeine ratios, and thus faster CYP1A2 enzyme activity. Participants administering insulin also showed faster CYP1A2 activity. While this relationship has previously not been directly assessed in humans, rodent models demonstrate that insulin induces CYP1A2 activity [5]. Moreover, an observational study in humans linked higher CYP1A2 activity with higher endogenous insulin levels [6]. These results further help to support the validity of our correction technique. Nevertheless, our technique needs to be validated in larger and stringently controlled samples, alongside comparison with systemic caffeine clearance data. If these future studies are successful, the correction could be applied, for example, in epidemiological settings where varied time frames between caffeine intake and saliva sampling are allowed. Here we used this correction only for data of participants who reported caffeine consumption within the time window of 3 to 12 h before saliva sampling. This is because, compared to the ‘gold standard’ approach of estimating CYP1A2 enzyme activity (systemic caffeine-to-paraxanthine clearance from blood) [9], the salivary paraxanthine/caffeine ratio only accurately reflects CYP1A2 activity during this time interval [14].

Interestingly, we found that the time interval between the final caffeine portion and saliva sampling was shorter in the patient group than in the control group. Thus, if both groups had equal CYP1A2 enzyme activity, a lower amount of caffeine would have been metabolized in the patients by the time of saliva sampling and a smaller salivary paraxanthine concentration should have been observed. By contrast, the paraxanthine concentration was higher in the type-2 diabetes participants, consistent with our conclusion that CYP1A2 activity was higher in the patients than in the controls.

The time-corrected paraxanthine/caffeine ratio was higher in study participants who reported higher caffeine consumption than the mean Swiss caffeine intake of 288 mg/day. Furthermore, statistical modelling revealed that high habitual caffeine intake was a significant predictor of faster CYP1A2 enzyme activity in our study sample. While type-2 diabetes status also contributed to the prediction of the paraxanthine/caffeine ratio, we suggest that out of the two predictor variables, caffeine intake was potentially the stronger mediator of faster caffeine metabolism. This is because caffeine is a known inducer of CYP1A2 activity [4, 26, 27], and the diabetes patients of the present study consumed larger amounts of caffeine (Table 1). Nineteen out of 57 patients administered insulin that has also been described as an inducer of CYP1A2 activity. When only type-2 diabetes patients were assessed, however, there were no significant differences in CYP1A2 activity between insulin users and non-users. This result suggests that insulin may not be a key driver of CYP1A2 activity in our study participants. Nevertheless, larger samples are needed in future studies to corroborate the existence of higher CYP1A2 activity in the type-2 diabetes patient population, and that this higher activity is due primarily to high caffeine intake.

We found no effect of age and BMI on CYP1A2 enzyme activity (Table 2). This finding was in-line with previous research [4]. Furthermore, there was no significant difference between CYP1A2 enzyme activity of participants who reported taking medication over the long-term, compared to participants that were not taking medications. This finding may reflect that medication has varying effects on CYP1A2 activity (inhibition, induction, or no effect), which are drug-specific [2]. Because we did not collect information regarding the specific medications of participants, it is impossible to further qualify this result.

In contrast to previous studies [4, 8], female gender, contraceptive pill use, smoking, and *CYP1A2* genotype also revealed no significant effect on CYP1A2 activity. Female gender has only a small influence on the paraxanthine/caffeine ratio [4], and our sample was probably not large enough to show a significant effect. In addition, the numbers of participants who reported taking oral contraceptives (*n* = 9) and smoking (*n* = 21), two fairly strong modulators of CYP1A2 activity [4], were low. These low participant numbers may explain why significant effects of these covariates were not seen. The paucity of smokers may also explain why, in the present data set, the *CYP1A2* -163 A > C genotype had no significant effect on CYP1A2 enzyme activity and speed of caffeine metabolism; since the more pronounced increase in CYP1A2 activity caused by this genetic variation is only observed in current smokers [7, 8].

The exact mechanism that links caffeine intake to speed of caffeine clearance is not yet fully understood. Animal studies have shown increased liver microsome CYP1A2 activity and mRNA levels in rats on very high doses of caffeine [26, 27]. This observation indicates an auto-induction of caffeine on CYP1A2 [4]. Support also comes from epidemiological studies, where a 1.45-fold higher CYP1A2 activity was observed per daily liter of coffee intake [2, 4]. Another suggestion is that persons with existing high CYP1A2 activity may consume more coffee because they metabolize it more quickly [28]. Coffee is a complex blend of organic compounds and therefore, constituent substances, aside from caffeine, may also contribute to its inducing effect [4]. For example, coffee beans are roasted at high temperatures, and thus may contain compounds similar to those found in tobacco smoke or chargrilled meats - known inducers of CYP1A2 activity [29]. Moreover, coffee’s diverse composition roots the existing controversy between coffee and caffeine consumption and risk of type-2 diabetes (see [30] for review). The limitations of this study include the reliance on self-reports to determine the timing of saliva sampling and the lack of information regarding habitual consumption of some dietary components known to influence CYP1A2 activity, e.g., chargrilled meat, as well as the intake of specific medications. Also habitual caffeine intake was measured by self-report questionnaire. While the validity of this method is established [12, 31], variability exists in the amount of caffeine per serving [32]. Therefore, caffeine use may have been under- or overestimated. The correction technique applied to the paraxanthine/caffeine ratios needs further, external validation. The fitted curve explained roughly 70 % of the variance in the Spigset data set [14], leaving 30 % unexplained. However, Fig. 1 suggests that this proportion of unexplained variance lies at time points greater than 12 h. We used the equation-derived paraxanthine/caffeine ratio at the 6 h time point. Finally, despite the regression model significantly predicting the paraxanthine/caffeine ratio, its explanatory capacity was low. This indicates that other, unknown or unmeasured predictor variables were also influencing CYP1A2 activity in our study sample. Previously, it has been noted that a large proportion of CYP1A2 activity is currently unexplained [2].

## Conclusions

In conclusion, while various factors probably influence CYP1A2 activity, high caffeine intake likely plays an important role. Here, we provide evidence that a positive association between caffeine consumption and CYP1A2 activity is present in our type-2 diabetes patient sample. Future studies are warranted to establish whether higher CYP1A2 enzyme activity is indeed causally related to high caffeine intake.

## Additional file

Additional file 1: Table S1. (Caffeine content of products available for consumption in German-speaking Switzerland) and experimental protocols (genomic assessment with salivary DNA; HPLC assessment of salivary caffeine and paraxanthine). (DOCX 39 kb)

## Abbreviations

BMI: Body mass index
BQL: Below quantification limit
CYP1A2: Enzyme cytochrome P450 1A2
*CYP1A2*: Gene cytochrome P450 1A2
HPLC: High performance liquid chromatography
SNP: Single nucleotide polymorphism
T2D: Type-2 diabetes
