# Supplementary material for: Assessment of CYP1A2 enzyme activity in relation to type-2 diabetes and habitual caffeine intake
Source: Nutr Metab (Lond). 2016 Oct 6;13:66. doi: 10.1186/s12986-016-0126-6 (PMC5052791; doi:10.1186/s12986-016-0126-6)
Supplement: Additional file 1: Table S1. — (Caffeine content of products available for consumption in German-speaking Switzerland) and experimental protocols (genomic assessment with salivary DNA; HPLC assessment of salivary caffeine and paraxanthine). (DOCX 39 kb) [file 12986_2016_126_MOESM1_ESM.docx]

***Higher CYP1A2 Enzyme Activity in Type-2 Diabetes Patients
Than in Non-Type-2 Diabetes Controls***

Emily Urry^1,2,3^, Alexander Jetter^4^, and Hans Peter Landolt^1,2^

**Additional File 1**

Revisions submitted to: ***Nutrition & Metabolism***

July 18, 2016

**Authors:**

Emily Urry (e-mail: [emily.urry@pharma.uzh.ch](mailto:emily.urry@pharma.uzh.ch))

Alexander Jetter (e-mail: Alexander.Jetter@usz.ch)

Hans Peter Landolt (e-mail: [landolt@pharma.uzh.ch](mailto:landolt@pharma.uzh.ch))

**Author for correspondence:**

Hans Peter Landolt, PhD

Institute of Pharmacology and Toxicology, University of Zürich

Winterthurerstrasse 190

8057 Zürich

Tel. +41-44-635-5953

Fax +41-44-635-5707

**Supplementary Table S1:** Caffeine content of products available for consumption in German-speaking Switzerland

| **Caffeine product** | **Size of serving (ml)** | **Total caffeine**  **per serving (mg)** | **Information source (website)** |
| --- | --- | --- | --- |
| **COFFEE** |  |  |  |
| Expresso-based coffee. Single shot (e.g. expresso, latte, cappuccino, mocha.) | 44 | 77 | Caffeine Informer ^a^ |
| Instant coffee | 240 | 57 | Caffeine Informer |
| Brewed/filter coffee | 240 | 107.5 | Caffeine Informer |
| Decaffeinated coffee | 240 | 4.5 | Caffeine Informer |
| **COLD COFFEE (‘Emmi cafe latte’)** |  |  |  |
| Cappuccino | 230 | 80 | Manufacturer |
| Caramel | 230 | 60 | Manufacturer |
| Expresso | 230 | 120 | Manufacturer |
| Light | 230 | 80 | Manufacturer |
| Macchiato | 230 | 80 | Manufacturer |
| Tahiti | 230 | 60 | Manufacturer |
| Zero | 230 | 110 | Manufacturer |
| **TEA** |  |  |  |
| Brewed/loose-leaf black tea | 240 | 48 | Caffeine Informer |
| Green tea/white tea | 240 | 25 | Caffeine Informer |
| **ENERGY DRINKS** |  |  |  |
| Redbull | 355 | 114 | Manufacturer |
| Redbull | 250 | 80 | Manufacturer |
| Redbull (sugar free) | 250 | 80 | Manufacturer |
| Migros own brand | 250 | 80 | Manufacturer |
| Migros own brand (sugar free) | 250 | 80 | Manufacturer |
| OK energy drink | 355 | 114 | Manufacturer |
| OK energy drink (light) | 250 | 80 | Manufacturer |
| Coop own brand | 250 | 75 | Manufacturer |
| Coop own brain (sugar free) | 250 | 75 | Manufacturer |
| Monster Energy | 500 | 160 | Manufacturer |
| Rockstar Energy Drink | 500 | 160 | Manufacturer |
| Lucozade | 380 | 46 | Manufacturer |
| Relentless Energy drink | 500 | 160 | Manufacturer |
| **SOFT DRINKS** |  |  |  |
| Coca cola, Pepsi, flavoured cola, shop-branded cola | 330 | 38 | Manufacturer |
| Coca cola, Pepsi, flavoured cola, shop-branded cola | 500 | 58 | Manufacturer |
| Diet coca cola, diet Pepsi, Coke Zero, Pepsi Max, shop-branded, diet flavoured cola | 330 | 38 | Manufacturer |
| Diet coca cola, diet Pepsi, Coke Zero, Pepsi Max, shop-branded, diet flavoured cola | 500 | 58 | Manufacturer |
| Caffeine-free Diet Coca Cola | 330 or 500 | 0 | Manufacturer |
| Dr Pepper | 330 | 38 | Manufacturer |
| Dr Pepper | 500 | 58 | Manufacturer |
| Iced tea (e.g. Lipton, Nestea) | 330 | 22 | Manufacturer |
| Iced tea (e.g. Lipton, Nestea) | 500 | 33 | Manufacturer |
| Iced tea (light/zero) | 330 | 22 | Manufacturer |
| Iced tea (light/zero) | 500 | 33 | Manufacturer |
| **DRINKING CHOCOLATE** |  |  |  |
| Hot chocolate (e.g. Suchard Express, Caotina) | 240 | 7 | Manufacturer |
| Cold chocolate (e.g. Nesquik, Micao, Comella) | 240 | 7 | Manufacturer |
| **SOLID CHOCOLATE** |  |  |  |
| Milk | 25g | 6.25 | Manufacturer |
| Dark | 25g | 17 | Manufacturer |
| **ICE CREAM** |  |  |  |
| Coffee-flavoured ice cream (e.g. Haagen-Dazs, Ben & Jerry) | 240 | 59 | Manufacturer |
| **CAFFEINE PILLS / SUPPLEMENTS** |  |  |  |
| Caffeine pills (e.g. ProPlus) | 2 tablets | 100 | Manufacturer |
| Supplements for general fatigue / lack of well-being (e.g. Tonikum D flüssig) | 10 | 0.9 | Manufacturer |
| **MEDICATION** |  |  |  |
| Cold and Flu (e.g. Rhinitin retard, Rhin-X) | 1 capsule | 25 | Manufacturer |
| Painkillers (e.g. Contra-Schmerz, Migrane-Kranit, Panadol Extra) | 500 mg | 65 | Manufacturer |
| Anti-nausea / motion sickness (e.g. Itinerol B6) | 2 capsules | 40 | Manufacturer |

Note: If there were several brands available for a given caffeine-containing product (e.g. Lipton and Nestea Iced Tea), the estimated caffeine content was averaged across the brands.

^a^ Caffeine informer website: http://www.caffeineinformer.com/the-caffeine-database.

**Experimental Protocols**

**Genomic assessment with salivary DNA**

Genomic DNA was extracted from saliva according to DNA Genotek’s instructions. Participants were genotyped for the cytochrome P450-1A2 gene (*CYP1A2*. -163C>A. SNP ID: rs762551. Assay ID: C_8881221_40) with TaqMan SNP Genotyping Assays (Applied Biosystems, Rotkreuz, Switzerland). Allele-specific polymerase chain reaction (PCR) was performed on a TaqMan thermal cycler (ABI PRISM^®^7900HT system; Life Technologies, Zug, Switzerland). The reaction volume contained 20 ng genomic DNA, 4 μl TaqMan Universal Master Mix (Applied Biosystems, Rotkreuz, Switzerland), 4 μl 20X SNP Genotyping Assay Mix, and 1.6 μl distilled H_2_O. Annealing temperature was set to 60°C. After running the PCR, an end‑point fluorescence measurement with the SDS 2.2 software package (Applied Biosystems, Rotkreuz, Switzerland) was obtained, to examine the samples and discriminate between the specific alleles. All genetic analyses were replicated at least once for independent confirmation of the results.

**HPLC assessment of salivary caffeine and paraxanthine**

The HPLC system consisted of a separations module equipped with a temperature-controlled autosampler (Alliance e2695 XC Separations Module, Waters, Dättwil, Switzerland) and a photodiode array UV detector (2998 PDA Detector, Waters). To summarize, a 225µl aliquot of saliva was prepared by addition of 75µl of trichloroacetic acid 20% containing the internal standard (100mg/l hydroxyethyltheophylline). After vortex mixing and centrifugation (2000g for 10 minutes at +4°C), 20µl of the supernatant were injected onto a Nucleosil 100 C18 reverse phase column (column dimensions 125 x 4mm; 5µm particle size; Macherey-Nagel, Oensingen, Switzerland) and eluted using a 4mmol/l acetic buffer (pH 4.0) containing 1% of acetonitrile, 1% of methanol, and 1.6% of tetrahydrofurane (v/v). The initial flow was increased from 0.8ml/min to 1.0ml/min within 2 minutes, and then kept stable at 1.0ml/min for 17 min, before initial conditions were restored after 2 additional minutes. The samples were usually analyzed in sets of twenty-five, with a calibration row before each set of participants’ samples, and a blank sample every ten unknown. Calibration was based on peak area ratios of paraxanthine and caffeine, respectively, over internal standard for ultraviolet absorption at 273nm and data point weighting by the inverse of concentrations. The lower limit of quantification (LLOQ) was 0.077ug/ml for caffeine and 0.024ug/ml for paraxanthine.
